# The interaction effects of the naked neck gene and housing system on egg production, egg quality, blood biochemical parameters and immunity of laying hens under hot climate

**DOI:** 10.5713/ab.24.0851

**Published:** 2025-06-10

**Authors:** Osama Abou-Emera, Ibrahim Al-Homidan, Gamal Rayan, Moataz Fathi

**Affiliations:** 1 Department of Animal and Poultry Production, College of Agriculture and Food, Qassim University, Al-Qassim, Saudi Arabia; 2 Animal Production Research Institute, Agriculture Research Centre, Giza, Egypt; 3 Department of Animal and Fish Production, College of Agricultural and Food Sciences, King Faisal University, Al-Ahsa, Saudi Arabia; 4 Department of Poultry Production, Faculty of Agriculture, Ain Shams University, Cairo, Egypt

**Keywords:** Animal Welfare, Cage System, Free-range, Genotype, Heat Stress

## Abstract

**Objective:**

Poultry producers use alternative housing arrangements, such as free-range systems, to allow birds to express their natural behaviors and to enhance consumer confidence in poultry products. Free-range systems provide hens with access to open spaces and better airflow, offering opportunities for thermoregulation through natural behaviors such as seeking shade or dust bathing. Compared to confined cage conditions, this can help reduce the negative effects of heat stress. In this study, we examined the productivity of laying hens segregated for the naked neck gene (Na) and raised in two distinct housing systems.

**Methods:**

A total of 540 laying hens, 24 weeks of age, were randomly assigned to a 2×3 factorial design consisting of three genotypes (NaNa, Nana, and nana) and two housing systems (wire cages and free-range pens). Productive performance, egg quality characteristics, immune response, and blood parameters were evaluated over a three-month period during the hot summer season.

**Results:**

The results showed that introducing the Na gene into chickens raised under hot climate conditions improved egg production, cell-mediated immunity, and eggshell strength. A significant reduction in blood cholesterol levels and the cumulative percentage of broken eggs was observed in naked neck genotypes (NaNa and Nana) compared to the normally feathered genotype (nana). Hens kept in cages produced significantly heavier eggs compared to those in the free-range system. Laying hens kept in a free-range system exhibited a higher relative yolk weight and an increased shell percentage. Additionally, a significant decrease in plasma cholesterol levels was found in layers raised in free-range systems compared to those in wire cages. Eggs produced by hens reared in free-range systems also had a darker yellow yolk color compared to those from caged hens.

**Conclusion:**

Introducing the Na gene into laying hens raised in free-range housing could be beneficial for improving egg production, immunity, and egg quality under high ambient temperatures.

## INTRODUCTION

The practice of raising chickens on littered floors or in free-range areas has gained interest due to its potential for producing organic products and allowing birds to exhibit natural behaviors. Although wire cages offer increased productivity and reduced labor requirements, they raise concerns regarding animal welfare [1,2]. There is no doubt that reducing the stocking density in free-range housing significantly alleviates the effects of heat stress by improving air circulation and enabling chickens to better regulate their body temperature. Currently, consumers prefer healthier and tastier eggs that are produced in accordance with animal welfare standards [3,4]. Several studies have demonstrated that the housing system can significantly influence egg production, egg quality, blood biochemical parameters, and immune responses in laying hens [5–7]. Since 2012, the European Union has prohibited the housing of chickens in traditional cages. Alternative rearing systems for laying hens, such as free-range, littered floor, and organic production have been accepted as replacements. By 2025, most retailers and food manufacturers in the United States have pledged to purchase only eggs produced on cage-free farms. Previous reports indicate that approximately two-thirds of consumers are willing to pay extra for organic animal products [8,9]. Similar to many countries in the Middle East, Saudi Arabia produces most commercial table eggs from laying hens housed in fully controlled houses equipped with conventional cage systems.

Due to the lack of sweat glands, birds are more sensitive to high temperatures than mammals. It is well known that elevated temperatures negatively affect both productive performance and immune responses in chickens [10,11]. In tropical countries, the Na gene may offer an effective solution to counteract the harmful effects of high ambient temperatures [12,13]. This gene reduces feather coverage in chickens by approximately 20%–30% in heterozygotes (Nana) and by 40% in homozygotes (NaNa), compared to their fully feathered (nana) counterparts [12]. As a result, naked neck birds exhibit greater tolerance and suffer less from heat stress. Heat shock protein 70 (HSP70), known for its rapid response to heat stress, is secreted to protect organs and cells from thermal damage. Birds carrying the Na gene show higher expression of the HSP70 gene compared to those with normal plumage [14]. Additionally, the Na gene reduces plucking costs and shortens the defeathering time during processing in slaughterhouses. In Saudi Arabia, several native chicken breeds, particularly those carrying the naked neck (Na) and frizzle (F) genes, are raised in various free-range systems for egg production [12]. Naked neck genotypes are gaining attention due to their superior adaptability to high ambient temperatures and greater disease resistance [15]. However, limited information is available on the productive performance and immune status of native breeds segregating for the Na gene when raised under different housing systems. Therefore, the objective of the current study was to assess the productivity of laying hens segregated for the naked neck gene (Na) and raised in two distinct housing systems under hot environmental conditions.

## MATERIALS AND METHODS

### Genotype, management, and housing system

The care and handling of the birds were conducted in accordance with the regulations of the animal ethics committee at Qassim University. A total of 540 laying hens, with a mean body weight (±standard error) of 1,173.6±14.4 g were used in the current experiment. The birds were randomly allocated in a 2×3 completely randomized design, consisting of two housing systems (wire cages and free-range pens) and three naked neck genotypes (NaNa, Nana, and nana). In the wire cage system, 180 hens were housed in 3-tier wire cages. Each group of four adjacent cages was considered one replicate (3 hens per cage). Each genotype had 5 replicates, totaling 60 hens per genotype. Each cage was equipped with a long feed trough and two nipple drinkers. In the free-range housing system, 360 hens were randomly placed into littered floor pens. Each genotype had 3 replicates, with 40 hens per replicate. All birds were initially raised on wood shavings from hatch until 18 weeks of age, after which they were either moved to wire cages or remained in free-range pens until the end of the experiment. Each free-range pen had an indoor area with access to a grass-covered outdoor area. The indoor area was part of an open-sided, naturally ventilated layer house and was equipped with four circular hanging feeders and two bell-shaped drinkers. The outdoor area also included two bell-shaped drinkers. Additionally, two nest box units (each with four cells) were placed in the indoor area of each pen. Hens were allowed to forage in the outdoor area during the day and sheltered indoors at night. Floor space in the caged system was 800 cm^2^ per bird, whereas the indoor free-range space was 2,000 cm^2^ per bird. Each free-range pen was also connected to a green outdoor area planted with alfalfa, providing 1.2 m^2^ per bird. Caged hens were given time to acclimate to their new environments and housing systems, starting from 18 weeks of age until the experiment began at 24 weeks. The experiment lasted for three months, from May to July. During the experimental period, the average indoor high and low temperatures were 35.3°C and 24°C, respectively, with relative humidity maintained at 55%. The lighting schedule was set to 16 hours of light and 8 hours of darkness. All hens were fed the same corn-soybean meal ration, containing 17% crude protein, 2,750 kcal/kg metabolizable energy, 3.6% calcium, and 0.4% available phosphorus. In both housing systems, drinking water was provided *ad libitum*, and feed was supplied daily at a fixed amount based on the birds’ productive age. Laying hens were vaccinated against Newcastle Disease Virus (NDV) at 24 and 34 weeks of age using the LaSota vaccine administered via drinking water.

### Laying performance and egg quality

Egg production and egg weight were recorded daily on a replicate basis. Stockpersons recorded the number of eggs with visible cracks or broken shells per replicate each day. In the final week of the experiment, 60 intact eggs from each genotype within each housing system were collected to assess internal and external egg quality characteristics [16]. All eggs were individually weighed to the nearest 0.01 g. Egg length and width were measured using a digital caliper, and the shape index was calculated as (width÷length)×100. Breaking strength for intact eggs was determined in kg/cm^2^ using an Egg Force Reader (Orka Food Technology). Egg weight and Haugh units were also measured automatically using an Egg Analyzer (Model # TRP-06) manufactured by Orka Food Technology. After separating the liquid contents, the eggshells, along with their membranes, were washed under running water to remove any adhering albumen. The wet eggshells were air-dried for 24 hours and then weighed to the nearest 0.01 g. The relative weight of the dried eggshell was calculated based on the total egg weight. Eggshell thickness (including membranes) was measured at three points on the shell (two poles and the equator) using a dial gauge micrometer (B.C. Ames Incorporated). The average of the three readings was calculated to the nearest 0.01 mm.

### Blood biochemical analysis

At the end of the experimental period, 10 blood samples from each genotype within each housing system were collected for blood biochemical analysis. Blood was drawn from the wing vein using heparinized anticoagulant tubes. Whole blood samples were centrifuged at 6,000×g for 15 minutes at 4°C to separate the plasma, which was then stored at −20°C until analysis. Plasma concentrations of total protein, albumin, total cholesterol, triglycerides, high-density lipoprotein (HDL), low-density lipoprotein (LDL), calcium (Ca), phosphorus (P), total antioxidant capacity (TAC), and malondialdehyde (MDA) were measured using a UV-visible spectrophotometer (PD-303UV; APEL). Commercial diagnostic kits (Stanbio Laboratory) were used according to the manufacturer’s instructions. Globulin levels were calculated as the difference between total protein and albumin [17].

### Immune assay

Heat stress negatively affects protein secretion and cellular function, including that of immune system cells. Accordingly, the cellular immune response was assessed using an *in vivo* cutaneous basophilic hypersensitivity test with phytohemagglutinin-P (PHA-P) (Sigma-Aldrich). Sixty laying hens were randomly assigned to this test (10 birds per subgroup). Each hen was intradermally injected in the right wattle with 100 μg of PHA-P dissolved in 0.1 mL of sterile saline. The thickness of the wattle was measured with a micrometer to the nearest 0.01 mm before injection (serving as the control measurement). Swelling at the injection site was then measured at 24, 48, and 72 hours post-injection to determine the response to the mitogen. The immune response was calculated as the difference between the wattle thickness before and after injection.

To evaluate humoral immunity, antibody titers against NDV in the serum of immunized hens were measured. Commercial ELISA kits (BioChek) were used in accordance with the manufacturer’s instructions [18].

### Statistical analysis

To assess the performance of laying hens carrying the Na gene raised in two different housing systems, a completely randomized experimental design was applied using a 3×2 factorial arrangement. The main factors were naked neck genotypes (NaNa, Nana, and nana) and housing system types (wire cages and free-range pens), along with their interaction. Statistical analysis was performed using the PROC GLM procedure of JMP ver. 11 (SAS Institute) [19]. To mitigate potential bias resulting from unequal group sizes, replicates were included as a random factor in the statistical model. All data are expressed as mean±standard error of the mean. Significant differences between groups were determined using Tukey’s test, with p<0.05 considered statistically significant.

## RESULTS

### Egg production and broken eggs

Table 1 illustrates the effect of genotype and housing system on egg production and cumulative broken eggs of laying hens. A significant increase (p<0.01) in egg production% was found in naked neck laying hens (NaNa and Nana) compared to normal feathered siblings (nana). Moreover, egg weight was significantly (p<0.01) affected by genotype, housing system, and their interaction. Based on the housing system, the laying hens kept in cages significantly produced (p<0.01) heavier egg weight compared to their counterparts raised in a free-range system. The presence of Na gene significantly improved egg weight irrespective of the type of housing system. Also, the interaction between genotype and housing system was highly significant (p<0.01). A significant increase (p = 0.05) in the percentage of cumulative broken eggs was detected in the normal feathered genotype (3.9%) compared to the naked neck genotypes (NaNa = 2.4% and Nana = 2.6%). In other words, the Na gene reduced cumulative broken eggs by about 33% to 38% for the NaNa and Nana genotypes, respectively, as compared to nana genotype. No significant difference was detected for cumulative broken eggs due to the effect of the housing system or the interaction between genotype and housing system.

### Egg quality traits

The results of egg quality traits as affected by genotype and housing system are shown in Table 2. Laying hens kept in a free-range system had significantly higher figures of yolk percentage, shell percentage, and yolk color score compared to those kept in cages. With respect to the Na gene effect, a highly significant difference between genotypes (p<0.01) for eggshell breaking strength and yolk color was detected. The naked neck genotypes (NaNa and Nana) recorded sronge eggshells (3.7 Kg/cm^2^) compared with those of normally feathered (nana) genotype (3.3 Kg/cm^2^). Also, a higher score of yolk color (4.0) was found for both naked neck genotypes compared to the normal one (3.2). No significant difference were noticed in all egg quality traits due to the effect of interaction between genotype and housing system.

### Cellular and humoral immunity

The effect of genotype and housing system on cellular and humoral immunity in laying hens is shown in Table 3. A significant improvement (p<0.05) in cell-mediated response was noticed for the Nana genotype after 24 h post-PHA-P injection (CMI = 0.78) compared to NaNa and nana genotypes (CMI = 0.63). Whereas, introducing the Na gene for laying hens in a homozygous or heterozygous state significantly (p<0.01) improved cellular immunity (CMI = 0.42) compared with their normal plumage counterparts (CMI = 0.16) after 72 h of injection. The homozygous naked neck (NaNa) was intermediate in all stages. In terms of humoral immune response, it could be noticed that there was no significant difference due to either genotype or housing system. However, a numerical increase was found in naked neck hens raised in wire cages. No significant interaction (G×HS) was detected in cellular and humoral immune responses.

### Biochemical, lipid profile, and antioxidant status

The effect of genotype and housing system on the blood plasma biochemical, lipid profile, and antioxidant status of laying hens is given in Table 4. No significant difference was detected for total protein, albumin, globulin, calcium, and phosphorus due to the main effects (genotype and housing system) or the interaction between them. The same trend was observed for antioxidant parameters (TAC and MDA). Concerning the blood lipid profile, it could be noticed that total cholesterol recorded significantly lower concentration (p = 0.048) in naked neck genotypes (NaNa and Nana). Laying hens kept in cages had a significantly (p = 0.041) higher total cholesterol concentration (140.7 mg/dL) compared to those kept in a free-range system (126.1 mg/dL). On the other hand, laying hens kept in a free-range system had a significantly (p = 0.044) higher LDL (39.1 mg/dL) compared to those kept in cages (34.9 mg/dL). In terms of antioxidant parameters, no significant difference was detected in TAC and MDA due to either genotype or housing system effect. Generally, as shown in Table 4, no significant differences for interaction between genotype and free-range system were found.

## DISCUSSION

**Figure 1:**
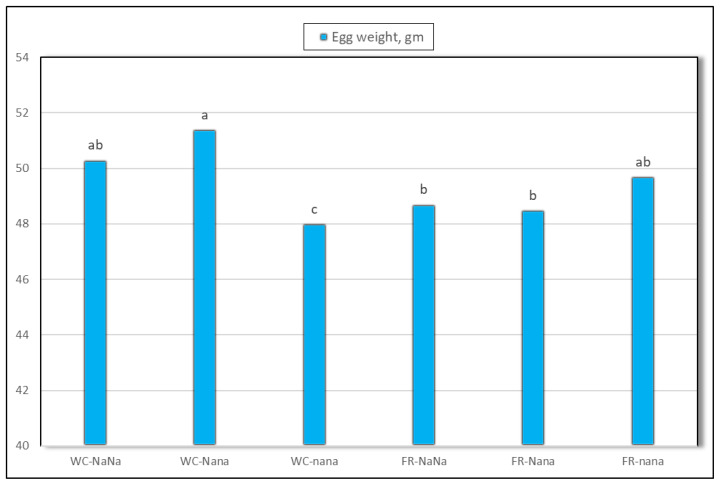
Effect of interaction between housing system (WC; FR) and genotype (NaNa; Nana; nana) on egg weight. a–c Different letters indicate significance at p<0.05. WC, wire cages; FR, free-range.

Egg production performance is one of the most economically important parameters in laying hens, influenced by both genetics and environmental factors. It is well established that a chicken’s performance can be negatively affected by uncomfortable housing conditions and high ambient temperatures. Given the growing consumer emphasis on animal welfare and the demand for organic poultry products, alternative housing environments must be considered, especially in hot climates. In our study, we did not observe a significant effect of the housing system on egg production. However, hens reared in free-range pens recorded a 2% higher egg production compared to those kept in cages. This increase may be attributed to additional feeding opportunities resulting from the presence of living plants, seeds, and insects in the outdoor environment. Previous studies have confirmed this assumption [20–22]. These studies reported that hens with outdoor access engaged in more foraging activities, such as scratching followed by ground-pecking. Nevertheless, numerous investigations have shown that laying hens housed in traditional cages tend to produce more eggs than those kept in alternative housing systems [23]. Conversely, other studies have reported that egg production is similar in laying hens raised in wire cages and cage-free systems [24,25]. Some studies have also indicated that introducing the *Na* gene into laying hens can increase egg production under high or moderate temperature conditions [14]. Egg weight is one of the most critical variables affecting egg components [26]. In the present experiment, egg weight increased by approximately 2% in hens kept in cages compared to those in the free-range system. However, previous studies have reported inconsistent results regarding egg weight. Some found that laying hens raised in cages produced larger eggs [27]. These findings suggest that housing systems influence egg weight differently, likely due to variations in management practices, nutrition, and environmental factors. Consistent with our results, higher egg weights have been reported in layers raised in conventional cages compared to those in cage-free aviaries or barns [7]. With respect to the effect of the Na gene, both genotypes of naked neck hens (NaNa and Nana) produced higher egg weights compared to the normally feathered genotype (nana). The improvement in egg weight may result from the reduction in feather coverage in naked neck birds, which allows more dietary protein to be allocated to egg production, thereby increasing both egg production and egg weight. Previous findings have confirmed this outcome, particularly under high ambient temperatures [28]. Improved thermal tolerance in Na hens may reduce physiological stress, contributing to better overall welfare. Furthermore, the significant interaction between the naked neck genotype and housing system allows producers to determine the most suitable genotype for a given housing system. Within the same housing type (conventional cages), naked neck laying hens (*NaNa*) produced significantly larger eggs compared to their normally feathered siblings (nana). In the free-range system, however, the different genotypes exhibited a similar trend in egg weight (Figure 1). These findings could inform breeding programs and housing strategies aimed at improving egg production while ensuring the welfare of hens under challenging environmental conditions. Cracked or broken eggs pose a serious problem and are a major source of economic losses in the egg production industry. Research has yielded varied results regarding the incidence of egg cracking in hens kept in different housing systems. A higher percentage of cracked eggs has been observed in wire cages compared to aviary or floor pen systems [29]. In our experiment, no significant difference was detected in the percentage of cumulative broken eggs due to the housing system. However, a numerical increase was observed in caged hens. Similarly, Voslárová et al [30] reported a higher proportion of cracked eggs in caged hens compared to those kept in floor pens. Regarding genotype, a notable reduction in cumulative broken eggs was observed in hens carrying the Na gene, whether in a homozygous or heterozygous state.

Several studies have shown that the Na gene significantly improves eggshell quality, which, in turn, reduces the incidence of broken eggs under high ambient temperatures [11,31]. Producing fewer broken eggs or higher-quality eggs translates to direct cost savings for farmers, thereby enhancing profitability. It is well documented that various factors influence egg quality, including genetics, nutrition, housing system, and environment [7,31]. Our results indicated that eggs produced by hens housed in a free-range system had a higher yolk percentage and greater eggshell proportion compared to those produced by caged hens. Moreover, yolk color intensity scored significantly higher in eggs from hens raised in the free-range system. Under moderate temperatures (22°C–26°C), Rizzi and Verdiglione [32] attributed the higher yolk percentage in free-range hens to increased dietary intake of grass and insects. Additionally, eggs from hens reared in free-range systems have been found to exhibit more intense yolk coloration compared to those from caged hens [6]. Hens housed in free-range pens also produced stronger eggs with greater shell thickness than those in wire cages [27]. The increased shell thickness and strength in free-range eggs may be due to reduced stress, as these hens can roam freely and engage in natural behaviors. Furthermore, exposure to natural daylight and greater motor activity in outdoor hens is thought to enhance mineral metabolism, leading to better mineral deposition in the eggshell [33]. According to Solomon [34], heat stress negatively affects eggshell thickness and breaking strength. In contrast, Haugh unit scores have been reported to be higher in eggs from caged hens compared to those from deep-litter systems [35]. Regarding genotype differences, our results revealed that the Na gene significantly improved yolk color score and eggshell strength, while also numerically increasing shell thickness compared to the normally feathered (nana) genotype. The improvement observed in naked neck chickens may be attributed to reduced feather coverage, which exposes more skin to solar radiation and increases vitamin D_3_ synthesis [36]. Another advantage of naked-neck hens is their ability to produce eggs with better shell quality under heat stress. This is likely due to limited respiration alkalosis compared to normally feathered hens, which increases the availability of free bicarbonate and calcium for eggshell formation and mineralization [12,14].

In the current study, neither the housing system nor the genotype had a significant effect on the humoral immune response. However, a numerical improvement in both cellular (p = 0.14) and humoral immunity (p = 0.24) was observed in hens raised in wire cages compared to those in the free-range system. This may be due to the higher risk of thermal stress in free-range systems compared to cages [37]. These results are supported by Rehman et al [5], who reported that birds in semi-intensive rearing systems exhibited higher antibody titers against the ND vaccine than those reared under free-range or confinement systems. Several studies have shown that chickens carrying the *Na* gene exhibit greater resistance to bacterial, viral, and parasitic infections compared to their normally feathered counterparts [14]. In tropical regions, naked neck birds have demonstrated lower mortality rates and higher disease resistance than those with normal plumage [38]. During heat stress, the physiological adjustments made to maintain body temperature can compromise immune function, increasing susceptibility to disease [14]. A recent study by Adenaike et al [39] concluded that Na birds exhibit more consistent and beneficial gut microbiota diversity, suggesting better gut health and improved immunity compared to normally feathered siblings. Regarding cellular immunity, our results revealed that the *Na* gene significantly enhanced the cell-mediated immune response compared to the normal feathered genotype, with this difference being most pronounced 72 hours after PHA-P injection. Numerous studies have shown that the presence of the *Na* gene, particularly in heterozygotes, significantly improves cell-mediated immunity under both low and high ambient temperatures [38,40]. The increased cellular immunity observed in Na genotypes may enhance disease resistance, which is especially critical in hot climates where heat stress typically weakens the immune system. Beyond reduced feathering, the Na gene has also been linked to elevated production of HSP70, a heat shock protein that mitigates the harmful effects of heat stress and supports immune function [14]. With respect to NDV antibody response, no significant differences were found among genotypes. In agreement with our results, Haunshi et al [41] reported no significant difference in humoral immunity (antibody response against SRBC) between heterozygous naked neck birds and their normally feathered counterparts. However, to enhance immunological insight, further studies are needed to determine cytokine profiles, particularly for key cytokines such as IFN-γ, IL-4, IL-10, and TNF-α.

Overall, the main factors (genotype and housing system) did not significantly affect most biochemical blood parameters. Several studies have reported no significant changes in total protein levels in the blood serum of chickens reared under different housing systems [5]. However, a significant reduction in plasma total cholesterol was observed in laying hens raised in a free-range system compared to those kept in conventional cages. This finding is consistent with the results of Zita et al [42], who reported a serum cholesterol concentration of 3.45 mmol/L in caged hens versus 2.80 mmol/L in littered-floor hens. The lower cholesterol level in hens from the free-range system may be attributed to increased physical activity and natural behaviors practiced in open environments. Pavlík et al [43] suggested a possible correlation between cholesterol concentrations in both blood and egg yolk, although some researchers have reported contradictory findings [39]. The concentration of LDL was significantly affected by the housing system (p = 0.044), with hens raised in free-range systems showing higher levels (39.1 mg/dL) than those in wire cages (34.9 mg/dL). Consistent with our results, Rehman et al [5] and DİktaŞ et al [44] confirmed that triglyceride levels did not differ significantly between housing systems. Regarding genotype effect, a significant reduction in total cholesterol was observed in naked neck hens (NaNa and Nana) compared to the normally feathered genotype (nana). Similar findings have been reported, indicating that cholesterol levels can vary by breed or strain [45]. In agreement with Rajkumar et al [46], the introduction of the Na gene in laying hens led to reduced levels of very-low-density and LDLs, while increasing HDL levels. The lower blood cholesterol content associated with the Na gene may be attributed to enhanced muscle development and greater egg production in naked neck genotypes compared to normally feathered birds. As for antioxidant parameters, no significant differences were found either among genotypes or between housing systems. However, further studies are needed to explore how the Na gene interacts with other housing systems (e.g., enriched cages, deep litter systems). Such insights could help optimize management practices to improve both productivity and animal welfare.

## CONCLUSION

In conclusion, both the naked neck (Na) gene and the housing system influence production performance, egg quality, and blood biochemical parameters. Specifically, under a free-range system, the introduction of the Na gene can improve egg production and egg weight, while also reducing cumulative broken eggs and plasma total cholesterol in hot climates. Additionally, the Na gene significantly enhanced the cellular immune response in laying hens housed in both wire cages and free-range systems. The presence of the Na gene also increased yolk color intensity across both housing types. Regarding the housing system, no significant difference was observed between the two systems in terms of egg production. Although overall production was unaffected by housing type, hens kept in cages produced heavier eggs. From a consumer preference perspective, free-range hens produced higher-quality eggs, characterized by greater relative yolk weight with darker yellow color, and higher shell percentage. Furthermore, the lower plasma cholesterol levels observed in layers raised in free-range systems, compared to those in cages, may reflect better welfare and health status. Regardless of the housing system, incorporating the Na gene into laying flocks is recommended under hot environmental conditions.

## Figures and Tables

**Table 1:** Effect of genotype and housing system on egg production and cumulative broken eggs of laying hens

Trait	Genotype (G)			HS		SEM	p-value		
		
	NaNa	Nana	nana	WC	FR		G	HS	G×HS
Egg production (%)	52.3^b^	56.0^a^	49.1^c^	51.9	53.0	0.46	<0.01	0.18	0.46
Egg weight (g)	49.6^a^	49.9^a^	48.9^b^	49.9^a^	49.0^b^	0.09	<0.01	<0.01	<0.01
Cumulative broken eggs (%)	2.4^b^	2.6^b^	3.9^a^	3.2	2.5	0.27	0.05	0.20	0.95

a–c Means of the same trait for each factor with different superscripts differ significantly (p≤0.05).

HS, housing system; WC, wire cages; FR, free-range; SEM, standard error of the mean.

**Table 2:** Egg quality traits as affected by Na gene and housing system of laying hens

Trait	Genotype (G)			HS		SEM	p-value		
		
	NaNa	Nana	nana	WC	FR		G	HS	G×HS
Egg shape index	75.3	75.4	75.8	75.6	75.4	0.19	0.54	0.75	0.22
Haugh units	60.9	59.4	58.1	59.2	59.8	0.55	0.16	0.60	0.20
Yolk (%)	35.0	35.4	35.5	34.9^b^	35.6^a^	0.15	0.32	0.05	0.15
Shell (%)	9.3	9.4	9.5	9.3^b^	9.6^a^	0.06	0.49	0.02	0.48
Yolk color (score)	4.0^a^	4.0^a^	3.2^b^	3.65^b^	3.85^a^	0.05	<0.01	0.04	0.48
Shell thickness (μm)	404.4	412.7	395.4	399.4	409.1	3.51	0.13	0.17	0.18
Eggshell strength (kg/cm^2^)	3.7^a^	3.7^a^	3.3^b^	3.5	3.6	0.04	<0.01	0.36	0.63

a,b Means of the same trait for each factor with different superscripts differ significantly (p≤0.05).

HS, housing system; WC, wire cages; FR, free-range; SEM, standard error of the mean.

**Table 3:** Effect of genotype and housing system on cellular and humoral immunity of laying hens

Trait	Genotype (G)			HS		SEM	p-value		
		
	NaNa	Nana	nana	WC	FR		G	HS	G×HS
CMI (h post injection)									
24	0.63^b^	0.78^a^	0.63^b^	0.71	0.65	0.032	0.05	0.33	0.41
48	0.49	0.61	0.47	0.55	0.50	0.028	0.08	0.35	0.49
72	0.28^b^	0.42^a^	0.16^c^	0.32	0.26	0.026	<0.01	0.14	0.35
Humoral immunity									
NDV titer	5,902.6	5,935.8	5,833.4	5,967.8	5,813.3	65.9	0.81	0.24	0.22

a–c Means of the same trait for each factor with different superscripts differ significantly (p≤0.05).

HS, housing system; WC, wire cages; FR, free-range; SEM, standard error of the mean; CMI, cell mediated index; NDV, Newcastle Disease Virus.

**Table 4:** Effect of genotype and housing system on biochemical, lipid profile and antioxidant status of laying hens

Trait	Genotype (G)			HS		SEM	p-value		
		
	NaNa	Nana	nana	WC	FR		G	HS	G×HS
Total protein (g/dL)	5.8	5.9	5.9	5.9	5.9	0.10	0.71	0.88	0.40
Albumin (g/dL)	4.2	3.7	4.2	4.2	3.9	0.10	0.09	0.14	0.83
Globulin (g/dL)	1.6	2.1	1.8	1.7	2.0	0.14	0.27	0.33	0.11
Ca (mg/dL)	27.2	25.3	24.5	25.6	25.8	0.66	0.22	0.92	0.41
P (mg/dL)	7.62	6.45	6.39	6.78	6.85	0.27	0.11	0.89	0.10
Blood lipid profile									
Total cholesterol (mg/dL)	129.4^b^	124.3^b^	145.5^a^	140.7^a^	126.1^b^	6.21	0.048	0.041	0.21
Triglycerides (mg/dL)	56.8	54.9	49.3	51.4	55.3	2.81	0.27	0.17	0.42
HDL (mg/dL)	68.4	86.1	66.7	65.4	83.6	5.11	0.53	0.18	0.72
LDL (mg/dL)	32.7	37.7	38.8	34.9^b^	39.1^a^	1.58	0.79	0.044	0.16
Antioxidant parameters									
TAC (mM/L)	1.40	1.33	1.35	1.40	1.31	0.05	0.96	0.40	0.82
MDA (mg/dL)	27.1	25.3	25.6	26.5	25.2	0.32	0.47	0.59	0.41

a,b Means of the same trait for each factor with different superscripts differ significantly (p≤0.05).

HS, housing system; WC, wire cages; FR, free-range; SEM, standard error of the mean; HDL, high-density lipoprotein; LDL, low-density lipoprotein; TAC, total antioxidant capacity; MDA, malondialdehyde.
